# A Comparative Evaluation of Magnetorheological Micropump Designs

**DOI:** 10.3390/mi13050764

**Published:** 2022-05-12

**Authors:** Sevki Cesmeci, Rubayet Hassan, Mahmoud Baniasadi

**Affiliations:** 1 Department of Mechanical Engineering, Georgia Southern University, Statesboro, GA 30460, USA; rh17100@georgiasouthern.edu; 2 Intel Corporation, Hillsboro, OR 97124, USA; mahmoud.baniasadi@intel.com

**Keywords:** micropump, magnetorheological, MRE, magneto-solid-fluid interaction

## Abstract

In this study, we assessed the performance characteristics of five different magnetorheological micropump designs, two of which were our proposed designs, while others were from the existing designs in the literature. Comparisons have been performed based on physics-based simulations, and the fully coupled magneto-solid-fluid interaction simulations were carried out in COMSOL Multiphysics software. For a fair and meaningful comparison, both the material and geometric properties were kept the same, and the simulations were run for one complete pumping cycle. The results showed that the proposed flap and duckbill valve models could pump 1.09 µL and 1.16 µL respectively in 1 s, which was more than the rest of the existing micropump models. Moreover, at 0.5 s, when the magnetic flux density was maximum, the flap and duckbill valve models could pump almost twice as fluid as some of the existing valve models did. The results also demonstrated that the flap and duckbill valve models were nearly five times faster than some of existing models. In conclusion, the proposed two micropump models could propel more net fluid volume than the existing micropump designs, experienced low leakage during the contraction and expansion phase, and had faster response times. We believe that the present study provides valuable insights for future micropump designs, which have an extensive range of application areas, ranging from insulin dosing systems for T1D patients to artificial organs to transport blood and from organ-on-chip applications to micro-cooling systems.

## 1. Introduction

With the recent advancements in manufacturing technologies, researchers have begun to explore novel designs at the micro and nano levels. Various micropump designs have been proposed and studied in the literature, including AC magnetohydrodynamic, piezoelectric, electroosmotic, acoustic, shape memory, thermo-pneumatic and so on. These designs have their advantages and disadvantages over one another in terms of scalability, biocompatibility, complexity, accuracy, cost and reliability. In this study, we focus on the magnetorheological micropumps. Magnetorheological elastomer (MRE) is a type of semi-active material, consisting of a rubber-like matrix with micron-sized iron particles embedded in it. MREs change their mechanical behavior under the influence of magnetic field. Their stiffness and damping properties alter with the intensity of the magnetic field. Due to this unique feature, they were utilized in various applications, ranging from vibration isolation systems in buildings and bridges to sensors for structural health monitoring applications and actuation systems such as micropumps [1,2,3,4]. There have been various MR micropump designs proposed in the literature in the last decade [5,6,7,8,9,10,11,12,13,14]. These designs usually varied based on the valves used. For example, Behrooz and Gordaninejad’s conceptual MR micropump design employed conical one-way valves to transport the flow unidirectionally [15,16], whereas Ehsani and Nejat investigated another conceptual design of an MR micropump, where they used one-way angled valves [17]. More recently, Xufeng et. al. proposed a magneto-active pulse pump (MAPP) with flexible check valves, resembling an eagle beak [18]. Researchers carried out fully coupled magneto-solid-fluid simulations to assess the performance of their proposed designs.

However, all of these designs were studied individually, and they are not compared against one another in terms of their pumping performances. In this study, we assessed the performance characteristics of five different MR micropump designs, two of which were our proposed designs, while others were from the existing designs in the literature. The models from the literature included the Behrooz, Ehsani and Xufeng [15,16,17,18] models. These were all the available magnetorheological micropumps with valves in the literature. Table 1 lists a brief overview of these pumps. All of these micropumps involved one-way valves with different designs. All included a flexible MRE top wall. While Behrooz and Ehsani models carried out fully coupled magneto-solid-fluid interaction simulations, Xufeng model did not. Behrooz and Ehsani models also carried out parametric studies to see the effects of important design parameters.

The other two models are the ones that are proposed in this study, namely the duckbill valve and flap valve models. Comparisons have been performed based on physics-based simulations, and the fully coupled magneto-solid-fluid interaction (MFSI) simulations were carried out in COMSOL Multiphysics software. For a fair and meaningful comparison, both the material and geometric properties were kept the same, and the simulations were run for one complete pumping cycle. The details of the design will be discussed in Section 2. Highly coupled MFSI simulations were carried out in COMSOL Multiphysics software (v5.6). The paper is organized as follows: the proposed design is discussed in Section 2, simulation methodology of five micropump designs is presented in Section 3 and summary and conclusions are discussed in Section 4.

## 2. Proposed Design

**Figure 1:**
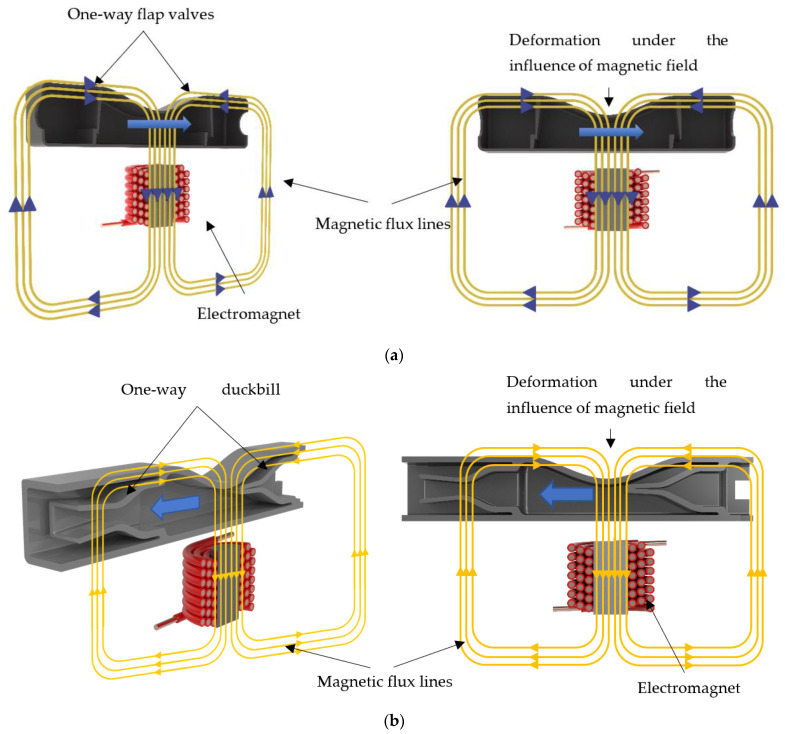
The proposed micropump designs with their main components: (**a**) flap valve model; and (**b**) duckbill valve model.

Among the proposed designs, the flap valve model consists of a pump chamber, two one-way flap valves and an electromagnet, while the duckbill valve model is composed of a pump chamber, two one-way duckbill valves and an electromagnet (Figure 1). The top wall of the pump chamber is made of a semi-active smart material called magnetorheological elastomer (MRE), while the rest of the structure is made from a passive elastomer. MREs falling in the category of composite materials are composed of a rubber-like base matrix, such as silicone and micron-sized iron particles doped in it. Because of their ferromagnetic properties, the MREs deform or can be designed to resist the deformation in the presence of a magnetic field, depending on design intent. Since their mechanical properties alter by the induced magnetic field, they are properly categorized as semi-active materials. In the proposed micropump design, under an external magnetic field, the top wall contracts inwardly in the pumping chamber. The amount of contraction depends on the intensity of the applied magnetic field, which can be controlled via an external electromagnet. The deformation on the top wall creates a squeezing effect on the fluid inside the flow chamber, pushing the fluid through the one-way valve in the front end. While this is occurring, a one-way flap and duckbill valve in the rear end prevents the fluid from leaking backward, thus creating an effective unidirectional pumping mechanism.

## 3. Simulation Methodology

### 3.1. Model Creation

**Figure 2:**
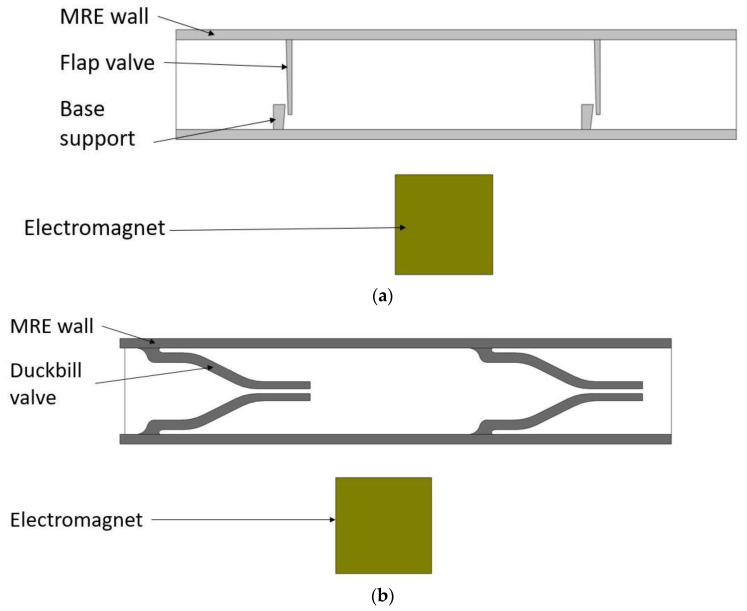
Micropump models with their main components: (**a**) flap valve model; and (**b**) duckbill valve model.

The proposed design involves coupled magneto-solid-fluid interaction physics. Thus, the performance of the pump could best be predicted with the help of computer simulations rather than simplified 1D analytical models. In this study, simulations were carried out using COMSOL Multiphysics software (v5.6, Burlington, MA, USA). Figure 2 shows a schematic of the model created in COMSOL.

**Figure 3:**
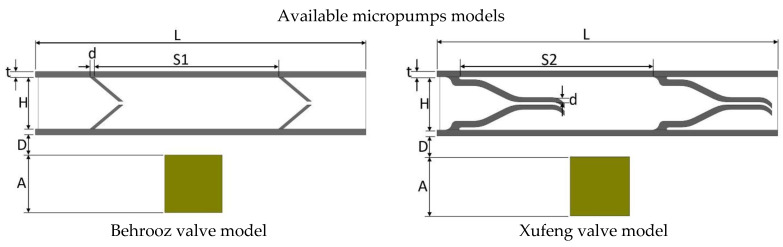

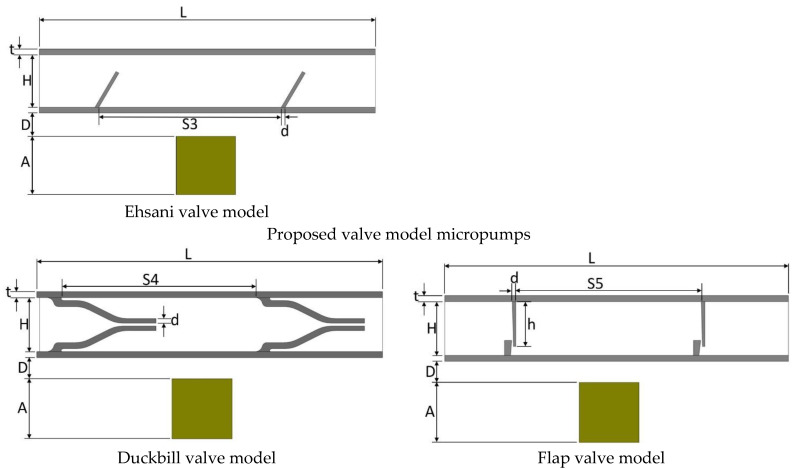
Valve models. Dimension values are given in Table 2.

The geometric and material properties of the 2D models are given in Table 2, while the dimensions are shown in Figure 3 for convenience. The magnet is placed 0.35 mm below the pump chamber. The height and wall thickness of the pump chamber is 1.10 mm and 0.10 mm, respectively, for all models. However, to maintain the same pumping area the distance between two valves is varied on different valve models. The elastic modulus of the pump material and average magnetic flux density acting on the upper wall are also kept identical, which are 1.2 MPa and 0.018 T, respectively.

The dimensions of the five 2D valve models are shown in the Figure 3.

**Figure 4:**
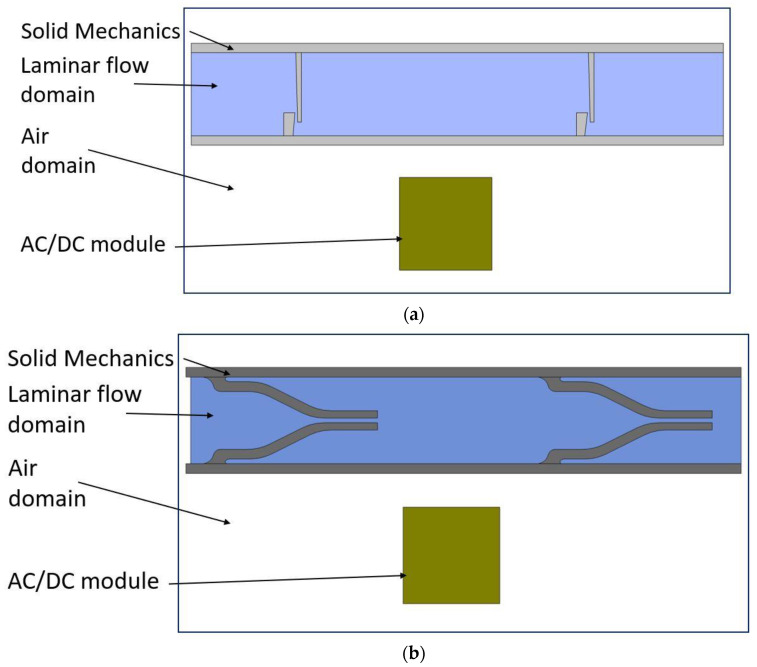
2D simulation model geometries: (**a**) flap valve model; and (**b**) duckbill valve model.

In this study, MRE is assumed as a ferromagnetic material that has constant structural and magnetic properties. The wall at the bottom does not deform in the presence of the magnetic field. It is assumed to be sitting on a flat rigid surface so the deformation on the top wall can activate the fluid flow. The magnetic field analysis was conducted in an AC/DC module, whereas the structural deformation of the pump chamber, including the top wall and one-way valves and fluid flow through pump chamber and valves were carried out in Solid Mechanics and Laminar Flow modules, respectively (Figure 4).

### 3.2. Simulation Procedure

**Figure 5:**
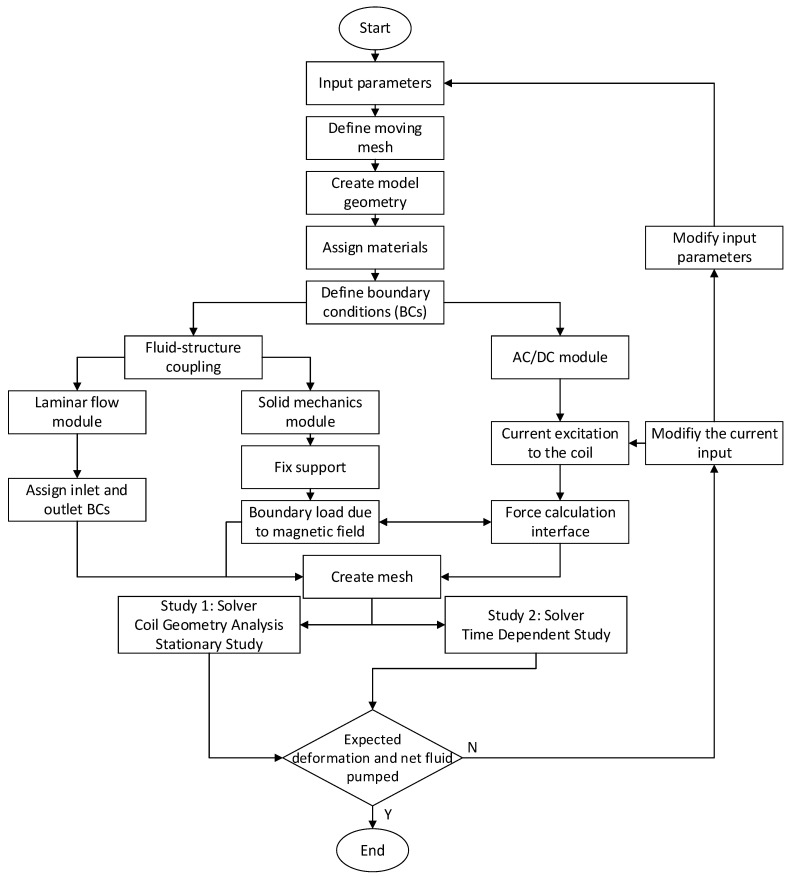
Flowchart of the simulation process in COMSOL.

The simulation procedure is illustrated with the flowchart shown in Figure 5. First, the variables such as geometric and material properties were defined. Since the simulation involves the deformation of the pump chamber and one-way valves, the moving mesh schemes were defined next. Then the simulation geometry was created, which was followed by the material assignment to all solid and fluid domains. Next, the respective boundary conditions were assigned in each physics module, i.e., AC/DC module, solid mechanics module and laminar flow module. Then these different modules were communicated with each other to transfer data between different flow physics. For example, the upper wall of the pump chamber deforms under the influence of a magnetic field since it is a ferromagnetic material. To model this phenomenon, the AC/DC module was run to calculate the magnetic field over the entire domain, including all solid and fluid domains. Then this information was passed to the solid mechanics module to calculate the resultant deformation under the influence of the magnetic field. Similarly, the deformation data on the seal was transferred to the fluid domain via fluid-structure coupling between the solid mechanics and laminar flow modules. This reflected itself as a pressure load on the fluid domain at the fluid-solid interface, causing the fluid flow, and thus the pumping effect. While the fluid was pushed through the front valve, the pressure data on the valves were communicated, again, with the fluid-structure coupling. Note that the fluid-structure coupling was a two-way coupling, which provided convenience when the data was communicated back and forth between the solid and fluid domains. This was done by defining fluid-structure interaction Multiphysics at all interfaces between the fluid and solid domains.

### 3.3. Boundary Conditions

As for the boundary conditions, a current excitation was added to the coil in the AC/DC module, and the force calculation interface was selected for the top edge of the upper wall of the pump chamber. This was to ensure that the Maxwell forces were transferred to the top edge for the desired deformation, and thus the resulting pumping effect. A sinusoidal time function and an external current density with a specific amplitude were applied to the core of the electromagnet. In the solid mechanics module, fixed support was added at the bottom wall of the pump chamber and the Maxwell surface stress tensor (magnetic interaction force between the pump chamber and magnetic field) was selected in the boundary load interface. In the laminar flow module, the pressures at the left and right outlets were set to 0 Pa (gage). This was to allow the fluid freely to pass in and out from two terminals. In addition, a no-slip condition was applied to the walls surrounding the flow chamber.

### 3.4. Computing Equations

Next, we discuss the governing equations for each physics involved. The following equations were solved for the magnetic domain

(1) ∇·J=0

(2) ∇×H=J

(3) $$B=\mu_{0}\mu_{r}H$$

(4) $$J=\nabla \times {\left(\mu_{r}\mu_{0}\right)}^{-1}B$$

where ∇ is the gradient operator, B is the magnetic flux density, J is the current density; $\mu_{0}$ and $\mu_{r}$ are the permeability of the vacuum and the relative magnetic permeability, respectively. The magnetic flux density H is determined in Equation (1), which is calculated from Ampere’s law. By combining Equations (1)–(3), the relation between J and B is obtained, which is given by Equation (4).

Moreover, Equation (5) shows the final equation of J

(5) $$J=\sigma E+\sigma v\times B+J_{e}$$

where σ is the electrical conductivity, E is the electric field, $J_{e}$ is the external current density and v is the velocity of the conductor. The magnetic flux density B and external current density $J_{e}$ can be calculated from Equations (6) and (7), respectively.

(6) B=∇×A

(7) $$J_{e}=\frac{I\cdot n}{a\cdot b}$$

In Equations (6) and (7), A is the magnetic vector potential, n is the number of turns of the coil of the electromagnet, I is the input electrical current to the coil and *a* and *b* are the cross-sectional dimensions of the core. The magnetic field density B can be calculated from Equation (8), when the external current density $J_{e}$ is known:

(8) $$J_{e}=\nabla \times {\left(\mu_{r}\mu_{0}\right)}^{-1}B-\sigma E-\sigma v\times B$$

The Maxwell stress is shown in Equations (9) and (10) the total stress, which is the result of magnetic stress and fluid pressure, is shown

(9) $$n\cdot \sigma_{maxwell}=-0.5n\left(H\cdot B\right)+\left(n\cdot H\right)B^{T}$$

(10) $$\sigma_{total}=\sigma_{maxwell}+\sigma_{p}$$

where n is the normal vector, $\sigma_{total}$ is the total stress, $\sigma_{Maxwell}$ is the Maxwell stress and $\sigma_{p}$ is the stress due to fluid pressure.

In the fluid domain, the flow is set to laminar incompressible flow appropriately. The following equations were solved in the fluid domain:

Steady continuity:

(11) $$\rho \nabla \cdot u_{fluid}=0$$

Navier-Stokes equations for stationary approach:

(12) $$\rho \left(u_{fluid}\cdot \nabla \right)u_{fluid}=\nabla \cdot \left[-pI+K\right]+F$$

Navier-Stokes equation for the time-dependent approach

(13) $$\rho \frac{\partial u_{fluid}}{\partial t}+\rho \left(u_{fluid}\cdot \nabla \right)u_{fluid}=\nabla \cdot \left[-pI+K\right]+F$$

where $u_{fluid}$ is the velocity vector, ρ is the density, p is the pressure, K is the turbulent kinetic energy and F is the volume force vector. The turbulent kinetic energy K can be determined from Equation (14).

(14) $$K=\mu \left(\nabla u_{fluid}+{\left(\nabla u_{fluid}\right)}^{T}\right)$$

For the solid mechanics, the following equations were solved:

For stationary approach:

(15) $$0=\nabla \cdot {\left(FS\right)}^{T}+Fv,\;F=l+\nabla u_{solid}$$

For the time-dependent approach:

(16) $$\rho \frac{\partial^{2}u_{solid}}{\partial t^{2}}=\nabla \cdot {\left(FS\right)}^{T}+Fv,F=l+\nabla u_{solid}$$

In Equations (15) and (16), Fv is the volume force vector, $u_{solid}$ is the displacement, *l* is the unit tensor and *FS* (*F* is the deformation gradient) is the first Piola-Kirchhoff stress tensor.

### 3.5. Grid Generation and Grid Independence Study

**Figure 6:**
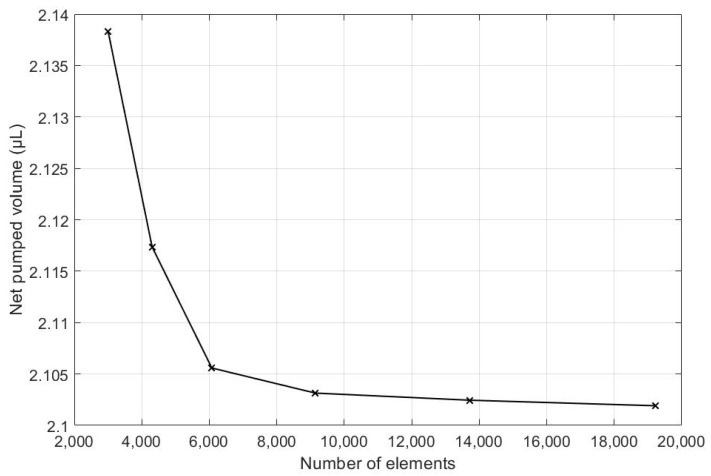
Net volume pumped is calculate for different number of elements.

As is commonplace procedure for conducting simulations in both Finite Element Analysis (FEA) and Computational Fluid Dynamics (CFD), a mesh independency analysis was conducted. To begin this process, a course mesh was applied to an initial simulation. Then, the mesh size was reduced continually, and the net pumped volume was monitored after each run. The mesh size reduction continued until there was no significant change between two sequential cases. This process is shown in Table 3, which is also visually illustrated in Figure 6. From the recorded cases, it can be seen that the percent change in the target parameter was 0.14% between grid numbers 4 and 5. Thus, grid number 4 is selected for the rest of the simulations.

### 3.6. Validity Study

**Figure 7:**
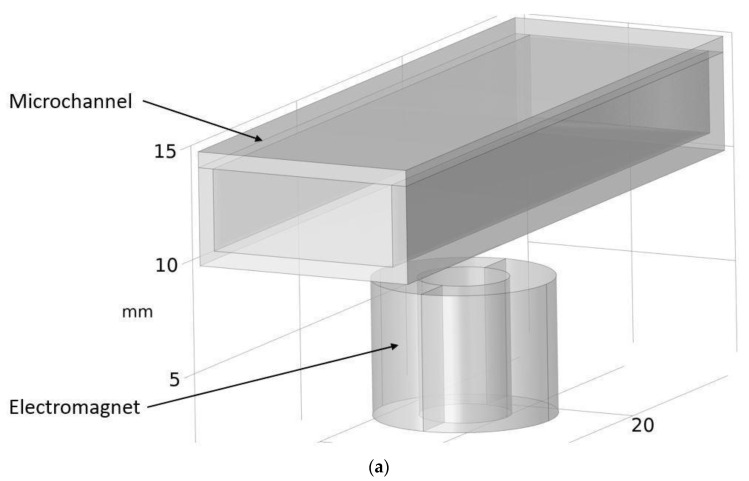

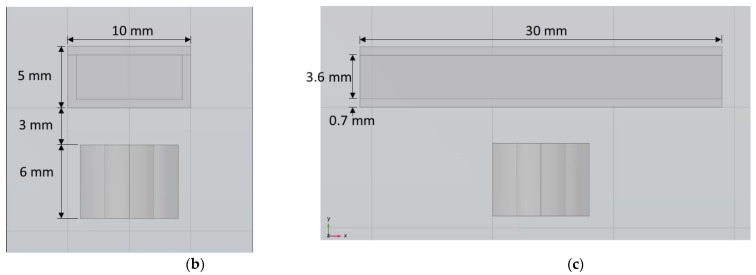
Schematic of the model studied in “Reprinted/adapted with permission from Ref. [18]. 2022, Rubayet Hassan”: (**a**) 3D view; (**b**) front view; and (**c**) side view.

Although COMSOL Multiphysics is a proven simulation tool and has been employed by thousands of scientific studies in the literature, it is always wise and scientifically required to validate the simulation approaches with the existing studies in literature when experimental data is not readily available. To this end, we selected the model presented in [18]. All parameters, boundary conditions, magnetic flux density and geometric dimensions were set to be the same. Figure 7 shows the model with the main components, as well as the dimensions.

**Figure 8:**
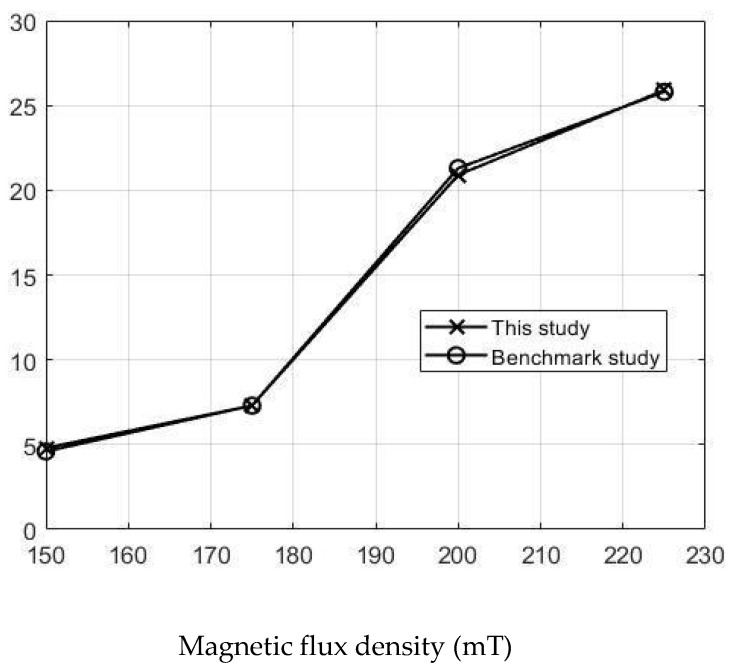
Net pumped volume comparisons between this study and benchmark study “Reprinted/adapted with permission from Ref. [18]. 2022, Rubayet Hassan”.

The simulations were carried out by following the procedure outlined in Figure 5. The comparisons between the benchmark study and this study are given in Table 4. From Table 4, it is seen that the upper wall displacement for both cases is the same for 75 mT, 145 mT and 175 mT magnetic flux densities. In addition to the deformations, volume flow rates were also compared. Figure 8 presents a comparison graph between the two cases. As seen from the figure, the volume flow rates matched closely between the two cases. Furthermore, the numerical data for the volume flow rates are presented in Table 5, with percent error margins. It can be seen that the average percent error between the two cases is about 1.6%, with minimum and maximum deviations being 0.00% and 4.16% at 150 mT and 175 mT, respectively. This validates our simulation methodology, allowing us to continue with the simulations of the pump designs.

### 3.7. Results and Discussions

**Figure 9:**
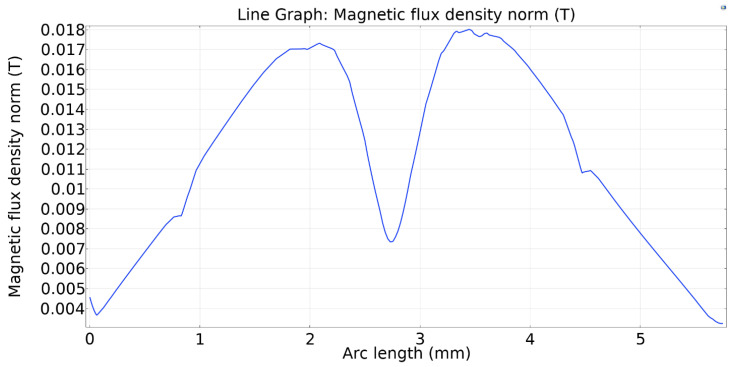
Magnetic flux density over the upper MRE wall under the magnetic field for the five valve models.

In this section, the pumping process is analyzed for one pumping cycle for the five micropump models. Comparisons are made in terms of volume flow rate and MRE wall response time. A full pumping cycle consists of a contraction phase, followed by an expansion phase. When the electromagnet is activated, the magnetic particles inside the MRE actuates and deforms the MRE wall downwards. The first phase of pumping is contraction. In this phase, the magnetic field over the MRE wall increase. Figure 9 represents the magnetic field of the upper MRE wall for all five models during the contraction period. The magnetic flux density is the same for the five models of micropump.

**Figure 10:**
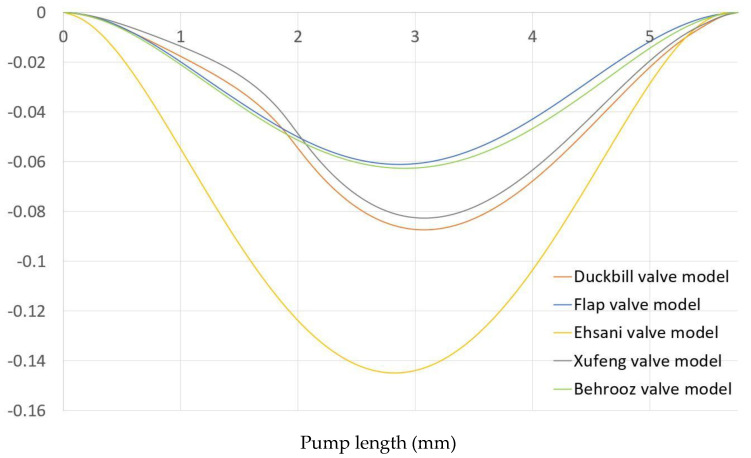
Vertical displacement of upper MRE wall for five different valve models.

Figure 10 shows the vertical displacement of upper wall for five different valve models. The Ehsani valve model experienced the largest upper wall deformation. This is because the distance between the upper wall and the upper tip of the valves is larger than other valves. That’s why the upper wall does not face any hindrance during deformation inwards. On the other hand, the MRE wall shows less deformation in the case of the Behrooz valve model. It is because the width of the Behrooz valve is more than the other valves, so the tips of the upper and lower valves sustain more resistance during the deformation of the MRE upper wall. The MRE wall bends due to the applied force. The deflection of MRE and the total applied force are reduced to zero when the expansion phase is completed. The fluid domain and solid parts are at rest initially for all five models.

When the upper wall begins to deform downwards it pushes the fluid inside the microchannel towards the two outlets. At this time, the fluid enclosed between the two valves deflects the left and right valves in a way that prevents the backflow, and propels the fluid to the right unidirectionally. This phenomenon is the same for all the five models.

**Figure 11:**
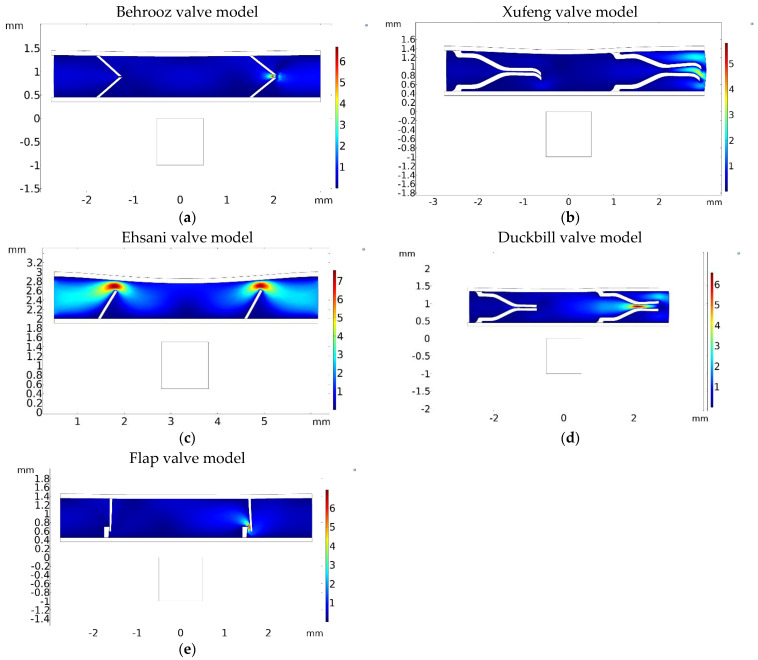
The velocity magnitude is shown for five different valve models. Maximum velocity magnitude at (**a**) *t* = 0.1 s for the Behrooz valve model, (**b**) *t* = 0.5 s for the Xufeng valve, (**c**) *t* = 0.5 s for the Ehsani valve, (**d**) *t* = 0.1 s for the Duckbill valve and (**e**) *t* = 0.1 s for Flap valve.

Figure 11 shows that the right valve is fully open when the fluid velocity is maximum at the right outlet, and at the same time, the left valve remains closed to prevent the back flow. This is the same for all five models.

**Figure 12:**
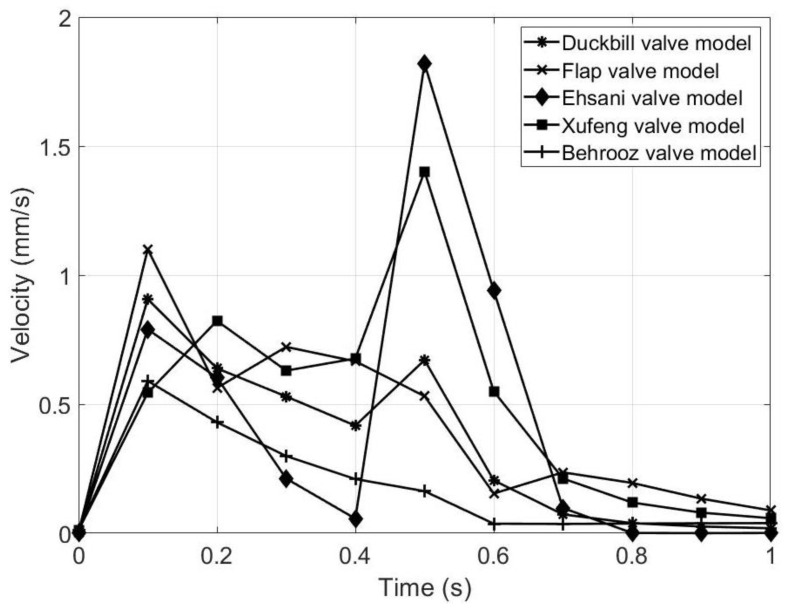
Graphical representation of velocity magnitude at the outlet of the five valve models.

From Table 6 and Figure 12, it is seen that the maximum velocity occurs from 0.1 to 0.5 s for different valve models, which means that the magnetic flux acting on the upper MRE wall rapidly. Among all, the Flap, Duckbill and Behrooz valves have the fastest response time, which is an advantage. In addition, the maximum velocity is obtained for the Ehsani valve model, but has a slower response time. Thus, in terms of rapid deformation of the upper wall, the proposed flap and duckbill valves model show the best performance.

**Figure 13:**
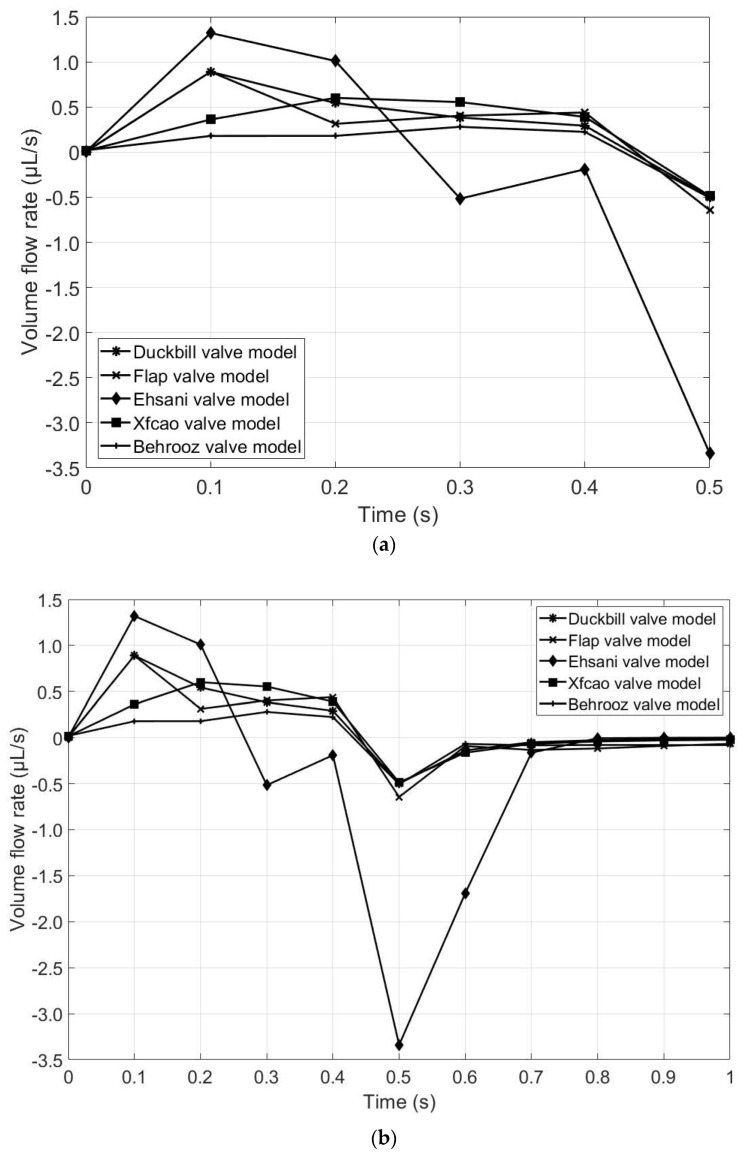
Comparison of volume flow rates for the five valve models: (**a**) contraction phase (until *t* = 0.5 s) and (**b**) both expansion and contraction phase (until *t* = 1 s).

Figure 13a represents the flow rate of the five valve models during the contraction phase. It is observed that the volume flow rate is high for the Ehsani valve model, which means that more fluid is propelled in the same amount of time compared to the other models. However, the Ehsani valve model is the only valve to have backflow during the contraction phase because of the large gap between the upper wall and the tip of the valves, which is a major disadvantage. Figure 13b represents the volume flow rate for both contraction and expansion phases. It is seen that all of the five models have backflow during the start of the expansion period (at *t* = 0.5 s), but the Ehsani valve model has the highest backflow compared to the other four models. Hence, the Ehsani model is the least advantageous among the five models. As seen from Figure 12, the Ehsani valve model shows the maximum velocity, but this maximum velocity is in the backward direction, which can also be confirmed by Figure 13b.

**Figure 14:**
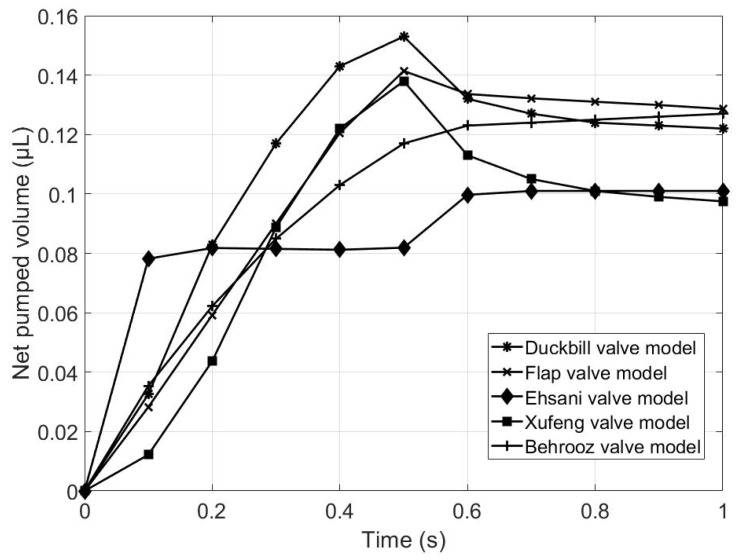
Comparison of net volume pumped.

Figure 14 and Table 7 demonstrate the net volume pumped by the five valve models during a 1 s pumping cycle. It is seen clearly from Table 7 that the duckbill valve model has the largest pumping capacity, followed by the flap valve model. It can thus be concluded that the proposed two valve models are superior to the other models in terms of net pumped volume during the same pumping cycle.

The material and the design of the chamber needed to be tuned to ensure the optimum deformation due to the electromagnetic force could be achieved, as stiff sidewalls would be a challenge for electromagnetic forces to be able to deform the chamber and increase the pressure to open the outlet valve. On the other hand, the return cycle needed to be as quick as possible to bring the chamber back to the initial shape ready for the next cycle, while generating enough suction to open the inlet valve and suck fluid into the chamber for the next pumping cycle. Both pressure and cycle time for pumping and suction cycles are directly related to the mechanical property of the elastic material and the design of the chamber. The thickness and design of the side walls has been tuned to optimize these pumping and suction performance of the pump. FEA analysis has been used to simulate the deformation and return of the chamber, to optimize the thickness and design of the chamber. While mechanical property of the chamber could be tuned by adjusting the UV-power density (power x exposure time) during the 3D printing process, a constant UV-power density (that was optimized for successful 3D printing of the thin wall for the specific elastic resin) has been used throughout all initial fabrication experiments.

The advantage of this fabrication is that geometry design and mechanical properties of the chamber could be easily modified and fine-tuned, with minimum limitation imposed from fabrication technique. The elastic resin used in this study is a new type of resin for 3D printing that has been developed by “Adaptive3D”.

## 4. Summary and Conclusions

In this work, we presented a comparative evaluation of several magnetorheological micropump designs. All designs were positive displacement pumps, pushing the fluid in one direction via two flexible valves. The Behrooz, Ehsani and Xufeng valves were existing in the literature, while the flap and duckbill valve models were proposed in this study. The performances of the pumps were evaluated through physics-based multiphysics computer simulations. The comparisons of the performance characteristics of the five pumps were presented and discussed.

The major takeaways from this study can be listed as follows:

- The comparison study revealed that:
  ○ the proposed duckbill and flap valve micropumps were five times faster than the Ehsani and Xufeng valve micropumps;
  ○ the backflow was comparatively less for the duckbill and flap valve micropumps, while the Ehsani models showed the maximum backflow;
  ○ the proposed flap and duckbill valve model could pump 1.09 µL and 1.16 µL in 1 s of pumping cycle, respectively, which is more than each of the three existing micropump models.
- After examining the MRE wall deformation, velocity magnitude and volume flow rate, it is concluded that the proposed duckbill and flap valve models can propel the largest amount fluid in the same time interval, and both of them have short response time to apply the magnetic field. Thus, in terms of the performance measures, it can be concluded that the proposed two models provide better results.
- The proposed study could be used for future MR micropump design considerations.

## Figures and Tables

**Table 1:** Existing magnetorheological micropump designs in the literature.

Micropump Models	Number of Electromagnets	Valve Type	Upper Wall Material	Parametric Study	Simulation
Behrooz valve model	1	One-way conical valve	MRE	Yes	MFSI
Xufneg valve model	1	Check valve	MRE	No	No
Ehsani valve model	1	One-way angle valve	MRE	Yes	MFSI

**Table 2:** Properties of microchannels.

Parameter	Symbol	Behrooz Valve Model	Xufeng Valve Model	Ehsani Valve Model	Duckbill Valve Model	Flap Valve Model
Height of the pump chamber	*H*	0.9 (mm)	0.9 (mm)	0.9 (mm)	0.9 (mm)	0.9 (mm)
Thickness of the upper wall	*t*	0.1 (mm)	0.1 (mm)	0.1 (mm)	0.1 (mm)	0.1 (mm)
Valve spacing distance	*S*	S1 = 3.079 (mm)	S2 = 3.238 (mm)	S3 = 3.069 (mm)	S4 = 3.204 (mm)	S5 = 3.102 (mm)
Length of micro channel	*L*	5.65 (mm)	5.65 (mm)	5.65 (mm)	5.65 (mm)	5.65 (mm)
Distance between the pump chamber and electromagnet	*D*	0.35 (mm)	0.35 (mm)	0.35 (mm)	0.35 (mm)	0.35 (mm)
Side of the electromagnet	*A*	1 (mm)	1 (mm)	1 (mm)	1 (mm)	1 (mm)
Magnetic flux density	*B*	0.018 (T)	0.018 (T)	0.018 (T)	0.018 (T)	0.018 (T)
Elastic modulus	*E*	1.2 (MPa)	1.2 (MPa)	1.2 (MPa)	1.2 (MPa)	1.2 (MPa)

**Table 3:** Net pumped volume for each grid number.

Grid Number	Number of Mesh Elements	Net Pumped Volume (µL)	The Difference in Net Pumped Volume (%)
1	3002	2.13832	-
2	4306	2.11735	0.98
3	6075	2.10558	0.56
4	9144	2.10314	0.51
5	13,708	2.10245	0.14
6	19,215	2.10191	0.11

**Table 4:** Comparisons of simulations between this study and benchmark study: (e)–(g) 3D shape of pipe under 75 mT, 145 mT, and 175 Mt during experimental study; (i)–(k) The deformation simulation corresponding to the experimental study in (e)–(g) “Reprinted/adapted with permission from Ref. [18]. 2022, Rubayet Hassan”.

Magnetic Flux Density (mT)	Benchmark Study ”Reprinted/Adapted with Permission from Ref. [18]. 2022, Rubayet Hassan”.	This Study	Maximum Displacement of Upper Wall (mm)
75	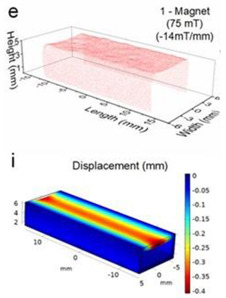	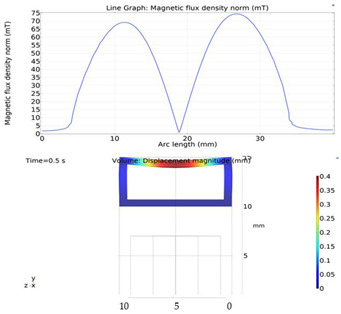	0.4
145	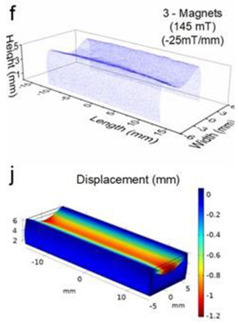	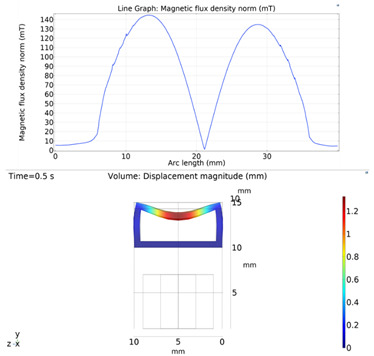	1.2
175	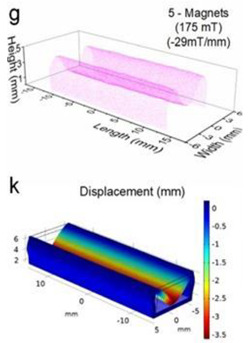	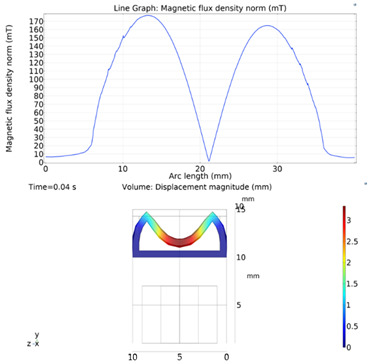	3.4

**Table 5:** Percent error of the flow rates between this study and benchmark study “Reprinted/adapted with permission from Ref. [18]. 2022, Rubayet Hassan”.

Magnetic Flux Density (mT)	Magnetic Flux Density (mT)	Flow Rate from Validation Case (µL/s)	Percentage of Error (%)
150	4.8	4.6	4.16
175	7.3	7.3	0
200	20.9	21.3	1.91
225	25.9	25.8	0.39

**Table 6:** Maximum velocity magnitude for all five valve models.

Valve Model	Maximum Velocity Occurred (s)	Maximum Velocity Magnitude at Outlet (mm/s)
Flap valve	0.1	1.10
Duckbill valve	0.1	0.91
Behrooz valve	0.1	0.59
Ehsani Valve	0.5	1.82
Xufeng valve	0.5	1.40

**Table 7:** Net pumped volume of micropumps.

Micropump Model	Net Pumped Volume (µL)
Duckbill valve model	1.16
Flap valve model	1.09
Xufeng valve model	0.92
Ehsani valve model	0.91
Behrooz valve model	1.03

## Data Availability

Not applicable.
